# An End-to-End Deep Learning Framework for Fault Detection in Marine Machinery

**DOI:** 10.3390/s24165310

**Published:** 2024-08-16

**Authors:** Spyros Rigas, Paraskevi Tzouveli, Stefanos Kollias

**Affiliations:** 1 Department of Digital Industry Technologies, School of Science, National and Kapodistrian University of Athens, 34400 Psachna, Greece; 2 School of Electrical & Computer Engineering, National Technical University of Athens, 15780 Athens, Greece; tpar@image.ntua.gr (P.T.); stefanos@cs.ntua.gr (S.K.)

**Keywords:** data collection, data engineering, deep learning, fault detection, marine IoT, MLOps, multivariate time series

## Abstract

The Industrial Internet of Things has enabled the integration and analysis of vast volumes of data across various industries, with the maritime sector being no exception. Advances in cloud computing and deep learning (DL) are continuously reshaping the industry, particularly in optimizing maritime operations such as Predictive Maintenance (PdM). In this study, we propose a novel DL-based framework focusing on the fault detection task of PdM in marine operations, leveraging time-series data from sensors installed on shipboard machinery. The framework is designed as a scalable and cost-efficient software solution, encompassing all stages from data collection and pre-processing at the edge to the deployment and lifecycle management of DL models. The proposed DL architecture utilizes Graph Attention Networks (GATs) to extract spatio-temporal information from the time-series data and provides explainable predictions through a feature-wise scoring mechanism. Additionally, a custom evaluation metric with real-world applicability is employed, prioritizing both prediction accuracy and the timeliness of fault identification. To demonstrate the effectiveness of our framework, we conduct experiments on three types of open-source datasets relevant to PdM: electrical data, bearing datasets, and data from water circulation experiments.

## 1. Introduction

The maritime sector plays a pivotal role in global trade, serving as a fundamental component of the international supply chain. Currently, international shipping carries over 80% of the world’s merchandise trade by volume, with maritime trade volumes projected to increase by 2.1% over the next few years [1]. Beyond its critical role in global commerce, the sector is also at the forefront of significant regulatory changes aimed at enhancing energy efficiency and reducing environmental impact. The International Maritime Organization (IMO) has introduced global measures [2], while the European Union has set ambitious targets to reduce ${\mathrm{CO}}_{2}$ emissions from shipping by 20–30% by 2030 [3]. These pressures, coupled with the inherent demands of the industry, necessitate continuous advancements in shipping operations. In response, the maritime sector is increasingly embracing digitalization in the era of Industry 4.0 [4], leveraging the exponential progress in fields like machine learning (ML) and cloud computing, enabled by the unprecedented volumes of available data [5].

Vessels cannot be studied in isolation from their external environment, and their operations are inherently complex, making it challenging to model their behavior solely based on traditional physical models. However, the widespread adoption of data-driven approaches like ML or deep learning (DL) has significantly advanced our ability to model and understand these complexities, surpassing the limitations of relying only on generic data such as ship particulars. Notable examples include but are not limited to using historical weather and navigation data to perform vessel route optimization [6,7]; collecting and analyzing vessel operational data to predict and minimize their fuel consumption [8,9]; and utilizing Automatic Identification System (AIS) data to predict emissions [10]. Another field where the accumulation of large volumes of data can be utilized to derive data-driven conclusions is Predictive Maintenance (PdM) of shipboard machinery, which stands out for its direct impact on both the operational reliability and efficiency of the vessel. By preventing unplanned machinery breakdowns, PdM not only generates significant cost savings but also increases the safety of the crew. This is particularly crucial considering that, according to a recent study [11], machinery damage or failure accounted for nearly half of all marine incidents globally in 2022.

In recent years, there has been a growing body of research focused on applying ML and DL techniques in the context of PdM in the maritime industry. Traditional ML algorithms, such as linear regression and non-linear principal component regression, have been utilized to characterize the condition of vessel engines [12] and model a ship’s hydrodynamic state [13], respectively. However, DL models based on neural network architectures have garnered increasing attention due to their ability to capture complex patterns in significantly large datasets. For instance, Multi-Layer Perceptrons (MLPs) have been employed to predict performance deterioration due to fouling [14] or propeller-hull degradation [15], and Convolutional Neural Networks (CNNs) have been trained to identify patterns on contour images indicating early signs of defective behavior in the vessel’s operational state [16]. Especially in the sub-domain of PdM known as fault detection, which is the primary focus of the present paper, substantial progress has been achieved through the use of neural network-based approaches. MLPs and Long Short-Term Memory (LSTM) models have been trained to detect faults in shipboard power systems [17], while fault detection relevant to bearing defects has been performed using multivariate LSTMs [18] as well as CNNs trained using transfer learning [19]. Additionally, CNNs have shown promising results in identifying faults in time-series data obtained from operational vessels [20], and LSTMs have been used alongside Variational Autoencoders (VAEs) to perform fault detection on (anonymized) sensor data obtained from maritime environments [21]. Finally, VAEs have also proven effective in fault detection tasks related to marine diesel engine degradation [22,23].

While the development of DL models for the aforementioned PdM tasks has seen growing interest in the past few years, acquiring the necessary data to power these models presents a significant challenge. Unlike routing or emissions monitoring, which can rely on satellite-provided AIS data [24], shipboard machinery poses a more complex problem. Traditionally, specialized personnel would be required to board vessels to manually gather measurements and assess the condition of onboard machinery, either directly or through subsequent analyses [25]. Moreover, Alarm Monitoring Systems (AMSs) on vessels typically restrict access to raw sensor data, providing only processed alarm outputs. Additionally, many pieces of equipment still rely on analog methods, such as gauge readings, which require manual inspection for data collection. Although some solutions have been proposed to improve data accessibility and integration from shipboard systems [25,26], the literature in this area remains in a nascent stage, delayed by 2–3 years compared to other industry sectors [27]. This underscores the need for the introduction of novel, comprehensive solutions analogous to those proposed and tested in other sectors of the industry [28,29,30], enabling the automated collection, integration, and processing of shipboard machinery data.

To this end, this paper proposes an end-to-end framework designed as a fully operational solution that manages the entire data lifecycle, from collection at the edge to the generation of actionable, data-driven insights. More specifically, the contributions of the current work can be summarized as follows:

- The introduction of a scalable edge server architecture that enables the collection and pre-processing of shipboard machinery data from sensors using the MQTT protocol.
- The construction of a cost-effective cloud pipeline that periodically ingests the collected data and performs computationally intensive operations, including model training.
- The development of a DL architecture based on Graph Attention Networks, along with the methodology to deploy multiple instances as ready-for-inference API endpoints.
- The introduction of a novel evaluation metric specifically designed to be applicable in real-world fault detection tasks.

The significance of this study for the maritime industry lies not only in the assembly of its components but also in the design of each element to meet specific criteria for real-world application. First and foremost, the framework attempts to strike a balance between deployment and computation costs, ensuring a high-quality and reliable solution using proven open-source libraries and packages. Moreover, it is built to be scalable, both horizontally and vertically, so that it can handle increasing amounts of data at the edge and adapt to varying operational demands in the cloud. The framework is also adaptable: in terms of sensors, a wide range can be accommodated as long as they support the MQTT protocol; in terms of the DL model, it can be applied to diverse data types and systems. Another key aspect is the emphasis on explainability in the architecture’s predictions, addressing a common limitation of DL [31] by enabling the quantification of feature importance, which is a strength traditionally associated with ML methods [13]. Importantly, understanding that the success of DL depends heavily on access to high-frequency, reliable data [32], the framework addresses two critical challenges faced in the maritime sector: the prevalence of proprietary, inaccessible datasets [31] and the scarcity of labeled fault data essential for model training [33]. Thanks to its reproducibility and ease of deployment, our framework allows users to begin curating valuable datasets, thus accelerating the progress of the field.

The remainder of this paper is structured as follows. Section 2 presents the proposed framework, detailing both the edge and cloud computing aspects, as well as the specifics of the DL architecture and machine learning operations (MLOps) practices, including the evaluation methods. Section 3 demonstrates the performance and explainability potential of the suggested framework and illustrates its capacity through experiments conducted on three different types of datasets relevant to PdM. Finally, Section 4 presents the conclusions drawn from these experiments and outlines potential directions for future research.

## 2. Proposed Framework

**Figure 1:**
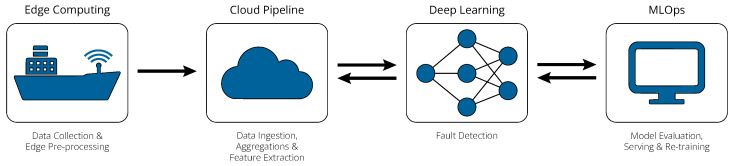
Visual representation of the framework decomposed into its four principal components.

As illustrated in Figure 1, the framework comprises four principal components. Initially, the edge component installed on the vessel is responsible for data collection from sensors and may also conduct preliminary data processing prior to cloud ingestion. Subsequently, the cloud pipeline orchestrates data ingestion and processes it within a multi-hop lakehouse architecture. Next, the deep learning component analyzes the processed data to detect anomalies in the time series. Lastly, the integration of robust MLOps practices handles the management of each model instance, i.e., model evaluation, serving, and iterative retraining. The purpose of this section is to describe each of these components in detail.

### 2.1. Edge Computing

To efficiently collect machinery data for further processing, one would either need AMSs equipped with custom APIs and adequate storage to extract raw data directly or deploy custom Data AcQuisition (DAQ) systems on the machinery. However, the former is a relatively new technology that has not been widely adopted, and the latter can be prohibitively expensive due to the costs associated with integrated sensor systems. Our framework is flexible enough to accommodate any scenario, from high-end DAQ and control systems to low-cost sensor-microprocessor systems designed to simply transmit raw measurements. The framework’s only requirement is that data must be transmitted using the MQTT protocol.

**Figure 2:**
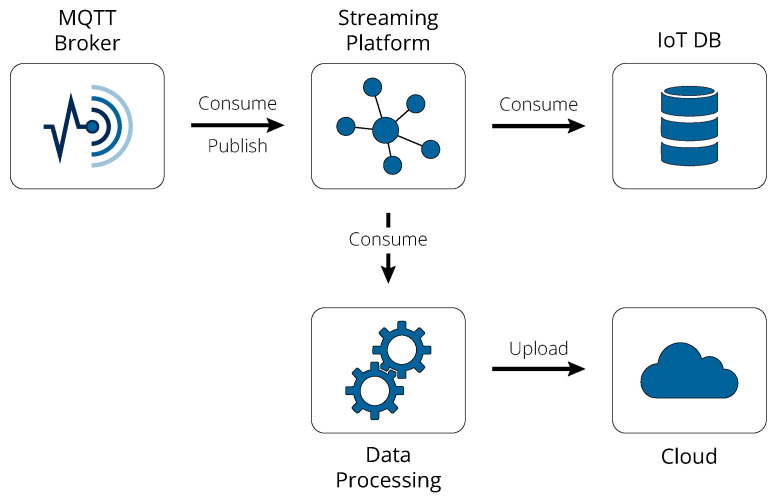
Schematic representation of the edge pipeline that handles data collection, pre-processing, and cloud upload.

MQTT was initially developed as a cost-effective solution for monitoring devices connected via satellite links, where communication costs were prohibitively high. Today, MQTT has become a widely adopted standard in the industrial IoT, valued for its lightweight publish-subscribe model and minimal energy consumption. Although its use in shipboard sensors has been limited so far, this is primarily due to the relatively recent adoption of the OASIS standard version, which was published only a decade ago. Running over TCP/IP, MQTT is particularly well suited for environments with unstable connections or limited network bandwidth, making it ideal for use on vessels. It operates by enabling devices (clients) to publish information to a central server (broker), which then distributes this information to other clients subscribed to specific topics. This method minimizes network bandwidth usage and reduces power consumption by not requiring continuous polling of data, making the protocol ideal for maritime operations, where the number of shipboard devices may be very large. In the proposed framework, the MQTT broker is the starting point of the edge pipeline, as illustrated in Figure 2, hosted on a standalone server PC or within the vessel’s existing server infrastructure. A detailed breakdown of this pipeline’s constituents follows.

The MQTT broker allows the shipboard machinery sensors to publish their data to preconfigured topics (henceforth, the term “sensor” will refer to the entire DAQ device, not just the signal detector). This setup offers flexibility in how topics are configured, allowing sensors to be logically grouped based on the needs of data consumption. For instance, sensors might be grouped by type, with all thermal sensors sharing one topic and electrical sensors another. Alternatively, sensors could be organized by their physical location, such as grouping all sensors located in the engine room under a single topic. However, a critical grouping criterion in the proposed framework is the frequency of the sensors’ measurements, which significantly influences the edge processing strategy. High-frequency data may require different preliminary processing at the edge to manage volume efficiently, while lower-frequency data can be processed more straightforwardly. Importantly, these grouping criteria are not mutually exclusive and can be combined to optimize data consumption by clients and subsequent processing. The proposed MQTT broker is Eclipse Mosquitto [34], due to it being lightweight and, most importantly, open-source; however, there is a plethora of other options [35].

The next major component of the edge pipeline is a distributed streaming platform deployed to manage and store the data streams provided by MQTT. Apache Kafka [36], widely recognized as the open-source industry standard for such workloads, is capable of handling a large volume of events with high throughput and low latency. By now, Kafka is so well established in the industry that its deployment is practically as straightforward as deploying a simple Docker container and writing some configuration files. Kafka also utilizes a publish-subscribe topic-based model; therefore, it might initially seem redundant in the same pipeline alongside MQTT. Nonetheless, it is crucial to understand that, despite their operational similarities, MQTT and Kafka serve distinct purposes. MQTT is primarily responsible for collecting data from various sources across potentially unstable network conditions. In contrast, Kafka excels at data storage and decoupling data collection from processing, enabling multiple consumers to access and process the same data streams independently.

Obviously, to facilitate the transfer of data from MQTT to Kafka, a bridge that acts as a consumer for the former and as a publisher for the latter is essential. This bridge can be custom-implemented, for example, using the Eclipse Paho [37] library, to meet specific operational needs such as data validation, timestamp addition, or the enhancement of data with additional features during ingestion. Alternatively, solutions like the open-source Telegraf [38] can be utilized to provide a pre-configured bridge, simplifying the integration process and reducing the need for extensive code development. Similar bridges are also required to consume data from Kafka, which are then either written directly to an IoT-optimized database or processed further before being uploaded to the cloud.

As for the first approach involving direct data writing, an IoT-optimized database such as InfluxDB or PostgreSQL with the TimescaleDB extension [39] can be utilized. Unlike Kafka, which is utilized primarily as a transient data layer with a short retention policy to minimize storage overhead, these databases are configured with a slightly longer retention policy. This extension is not intended for analysis but to ensure that recent data are readily accessible for providing a critical backup in case of severe data flow disruptions. Additionally, by connecting the IoT database to dashboard applications like Grafana—often paired with InfluxDB for sensor data monitoring [40]—engineers on board can perform visual checks to confirm that sensors are online and functioning correctly.

When it comes to the pathway that involves the upload of the data to the cloud, there exists an option to perform pre-processing at the edge before transmission. This step is particularly common when dealing with high-frequency sensors, where continuously uploading large volumes of data is not feasible. In such instances, necessary pre-processing, such as extracting fine-grained features essential for cloud-based analysis, is executed at the edge. Basic pre-processing tasks like aggregations or simple transformations can be handled efficiently using Kafka Streams. For more complex pre-processing requirements, custom services leveraging technologies like Pandas [41] for data manipulation and Dask [42] for parallelization and lazy evaluations may be employed. In scenarios with substantial computational resources available at the edge, Apache Spark [43] can be utilized as the most efficient and scalable solution. Similar to Kafka, simply allocating more resources allows the parallelization of tasks or the execution of more intensive workloads. Regardless of the choice for pre-processing, the data are eventually uploaded to the cloud. Here, there are two main options: batch versus streaming uploads. Given the nature of maritime operations, where equipment failures typically develop over weeks or months rather than instantaneously, regular batch uploads via HTTP at pre-determined intervals or during hours of low average network usage should generally be sufficient and preferred. This also minimizes prolonged network use, which is beneficial given the often unreliable connectivity at sea. In instances where an internet connection is temporarily unavailable, such as when a vessel is traveling through remote regions, the upload process can be configured with periodical retry mechanisms. This setup allows data to be transmitted as soon as connectivity is restored, without disrupting data pipelines or risking data loss.

The proposed edge architecture is designed to be highly scalable and adaptable, capable of operating on hardware ranging from low-cost PCs to more complex server clusters, as long as such infrastructure is available on the vessel. In environments where server clusters are used, Kafka’s distributed nature can be fully exploited. Via horizontal scaling, it is straightforward to deploy more Kafka instances and partition the data that each instance processes. Additionally, more Kafka instances can be deployed as redundancies to ensure higher data availability. With more computational resources available, tools like Spark can also be utilized for seamless pre-processing. However, all of these are simply conveniences rather than requirements. Notably, the pipeline is also sensor-agnostic in the sense that it requires only that data be transmitted via MQTT, accommodating everything from high-end DAQs to basic low-cost sensors. This flexibility even allows for simulated control scenarios without requiring sensors to be specifically configured for them. For instance, in scenarios requiring high-frequency data collection at intervals rather than continuous monitoring, the system can dynamically subscribe to relevant topics for the duration needed to gather the necessary data, thereby optimizing resource use without depending on the sensors’ hardware. With such capabilities at the edge, one might question the need for cloud involvement at all. However, the cloud plays a critical role, not only in ensuring long-term data storage but also in deploying DL models that are trained on the data to provide essential insights. Due to their computational requirements, these models are less suited to run on the edge in marine environments, especially given their constraints on power usage and the impracticality of preemptively scaling physical infrastructure. Thus, transitioning to cloud-based processing not only addresses these limitations but also enhances the overall efficacy and scalability of the proposed framework.

### 2.2. Cloud Pipeline

Following ingestion, the data are stored within a lakehouse architecture [26], which combines the expansive storage of a data lake with the structured query capabilities of a data warehouse. Within this lakehouse, processing and transformations are performed to extract fine-grained features to be utilized for DL. These features are used in subsequent DL experiments, including the training and evaluation of predictive models used for inference. This initiates the MLOps cycle, which involves continuous model retraining and evaluation to maintain model efficiency and relevance. A platform that facilitates all these processes as a unified ecosystem in a computationally optimized and scalable manner is Databricks [44], which is integrated across most major cloud providers.

At this point, it is important to address an apparent contradiction regarding the design of a low-cost solution alongside the use of what are often perceived as expensive cloud services. In reality, the ongoing costs associated with the staffing, maintenance, and scaling of on-premises systems often surpass the costs of cloud services, particularly when also considering the requirements related to physical space and power consumption. Additionally, platforms like Databricks provide dynamic scaling and efficient resource allocation, reducing the need for extensive in-house expertise. Moreover, cloud providers offer guarantees regarding data security, which is critical in the competitive maritime industry where stakeholders are highly protective of their data. As discussed in the following sections, very few maritime-related datasets are open and this is one of the main reasons. It thus becomes evident that cloud platforms align with the framework’s commitment to cost-efficiency, particularly over the long term. With these considerations in mind, in the remainder of this subsection, we describe how the data pipeline works using Databricks as the core platform.

Databricks implements the lakehouse architecture through its integration with Delta Lake [45], which is an open-source storage layer built on top of existing cloud storage services (e.g., ADLS2 for Microsoft Azure, S3 for AWS, and Storage for GCP). Among the main features that Delta Lake brings to traditional data lakes are ACID transactions, data time travel, scalable metadata handling, and schema enforcement and evolution. Moreover, Databricks incorporates Apache Spark in its runtime environments. This integration enables the optimized and parallel execution of code, as well as the distribution of data across multiple nodes, which is essential for handling a large volume of data from multiple sources.

**Figure 3:**
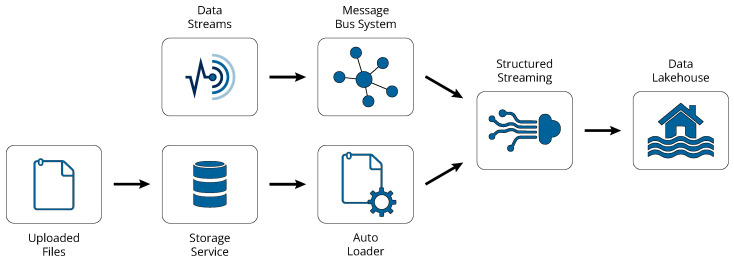
Schematic diagram displaying the data pipeline, which runs on the cloud to ingest and process the IoT data arriving from vessels as streams or batches.

The pipeline depicted in Figure 3 starts with the ingestion of the sensors’ telemetry, either pre-processed at the edge or not. If the data arrive online in streams, a message bus service (e.g., Event Hubs for Microsoft Azure, Kinesis for AWS, and Pub/Sub for GCP) is necessary to serve as a buffer, bridging the data transmission at the vessel with the data ingestion from the data pipeline. On the other hand, in batch-upload scenarios, a storage service acts as the staging area where the data are received as files, preferably in read-optimized formats like Apache Parquet [46]. To incrementally ingest the new data files as they arrive in the storage service, Databricks’ built-in Auto Loader is utilized. The Auto Loader maintains an internal state to track all processed files, thus ensuring that no file is missed while also offering features such as schema inference and handling of schema drift.

Subsequently, the data are written into tables in the lakehouse using Spark’s Structured Streaming [47]. Structured Streaming is an engine built on top of Spark which enables the ingestion of data from a source to a sink via built-in connectors, using a high-level API. In the case of data from incoming streams, the source is the message bus service used, while in the case of uploaded files, the source corresponds to the storage service monitored by the Auto Loader. Finally, in both cases, the sink connector points to the Delta Lake.

**Figure 4:**
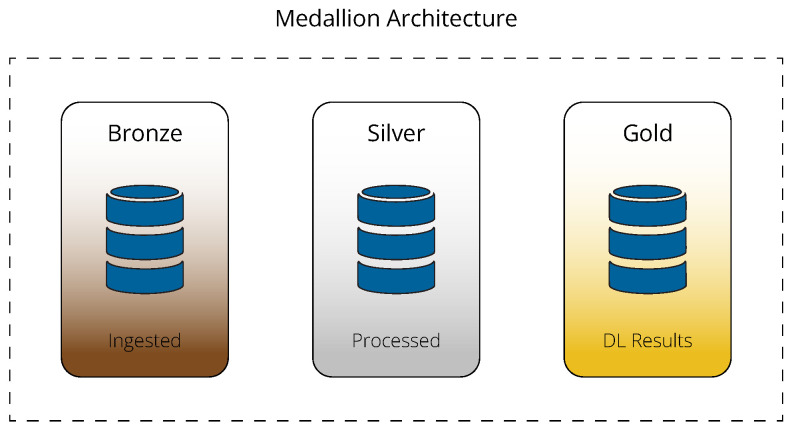
Illustration of the medallion architecture and its three layers.

The architecture chosen for the Delta Lake in our framework is known as a multi-hop or medallion architecture and is illustrated in Figure 4. The idea behind this design is to organize the data into distinct layers within the lakehouse, with the structure and quality of the data being progressively enhanced as they move through the layers. The number of layers is flexible and is based on specific operational needs or data management strategies. For the purposes of the presented framework, a three-layer configuration is recommended. The initial layer, known as the bronze layer, contains tables that hold the raw data ingested via the aforementioned procedure.

Once the data are successfully ingested into the bronze layer, they undergo a refinement process to prepare them for DL applications. This process involves extracting feature vectors, which are then stored in the middle layer of the lakehouse, known as the silver layer. As discussed in the following subsection, the DL model processes multivariate time series, which are measured from various sensors installed on a single machine. Therefore, the first step of this refinement process involves mapping sensor-level data to system-level data, i.e., combining univariate time series to create a multivariate one. If the data have not been pre-processed at the edge, this typically includes aggregations (e.g., averaging or extracting median values) to ensure that the length of the univariate time series is consistent across all sensors installed on a machine without resorting to methods like padding. Further data processing might involve transformations such as min-max normalization or standardization using pre-trained scalers. This implies that historical data are available on which these scalers have been trained. All of these operations are performed using Spark, mainly to ensure short execution times and to handle data regardless of volume. It is worth noting that cleaning and filling missing values is not necessary, as it has already been handled at the edge. For example, in case a sensor malfunctions and subsequently stops operating, it will stop transmitting signals, appearing as if it has been disconnected from the DAQ’s corresponding channel. The DAQ can then transmit a pre-specified value to indicate that the sensor was disconnected, and these values can be filtered out or replaced by the last correctly measured value once they reach the onboard server.

The fine-grained data from the silver layer’s tables are input into DL models with the aim of generating insights regarding the condition of monitored devices through fault detection. Of course, the framework is versatile, allowing for the integration of “traditional” statistical or threshold-based methods, either independently or in conjunction with DL models. The results from these analyses are stored in the final layer of the lakehouse, known as the gold layer, which is primarily designated for reporting purposes. Consequently, the data in this layer are consumption-ready and tailored for end-use applications. To consume these data, a dashboard application can be implemented, leveraging the numerous connectors supported by the Delta Sharing protocol—an open protocol that allows sharing data that live in a Delta Lake without having to copy them to another system [48]. This dashboard can be configured with a role-based access control model to maximize its business utility. For instance, stakeholders might have access to high-level metrics, such as the number of faults detected in each vessel of their fleet. On the other hand, engineers on board may be able to investigate not only which data points of a time series are identified as faults by the models but also why, as long as the deployed models are explainable.

Before proceeding to the proposed DL architecture for fault detection, it is important to highlight that Databricks not only manages the execution of the aforementioned operations but also orchestrates the entire workflow through Databricks Workflows. The arrival of new data, whether in file or stream form, automatically triggers the workflow, with various notebooks designated to handle different aspects of the data pipeline. Nonetheless, for use cases requiring broader integration capabilities, such as triggering external functions via APIs or interfacing with other cloud services, one may consider switching to a dedicated pipeline management service capable of running Databricks notebooks as jobs (Data Factory for Microsoft Azure, Glue for AWS, and Dataflow for GCP).

### 2.3. Deep Learning Architecture

As previously mentioned, the framework can operate using “traditional” analysis methods. However, marine environments present unique challenges that can limit the effectiveness of these techniques. If one takes vibration analysis via Fourier transformations as an example, which has been extensively employed for machine condition monitoring [49], numerous problems immediately become apparent: variable background noise is always present because no machine on a vessel operates in isolation; machinery often runs under alternating load conditions, which can lead to non-stationary signals; and marine equipment is frequently subject to transient events, such as sudden changes in engine speed or impacts from waves, which can introduce short-lived changes in vibration signals, to name a few. To effectively address these issues, extensive experimentation is necessary—sometimes individually for each piece of equipment. This might involve setting baselines during dry dock measurements or capturing signals under varying operational conditions and then performing extrapolations. Given these complexities, maritime operations are ideal candidates for the application of DL methods, as they do not require such extensive preliminary steps, relying instead on a data-driven approach that adapts to the inherent variability of the environment. However, when available, such information is very valuable for data pre-processing, as it may enhance the model’s performance.

**Figure 5:**
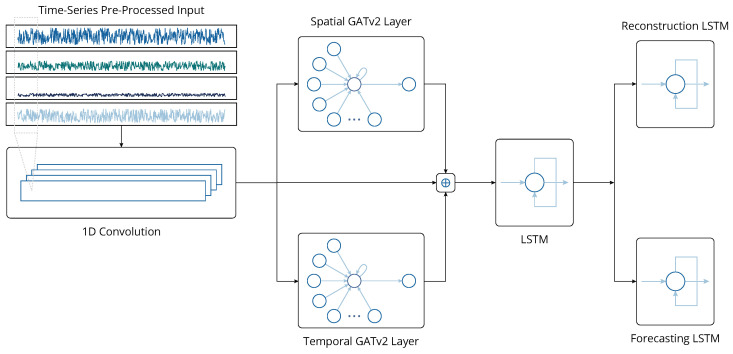
Diagram of the GAT-based DL architecture for fault detection.

Naturally, the choice of a suitable DL architecture is not trivial since DL models are not one-size-fits-all solutions by default. Prior to the development of the DL architecture, specific criteria were established. Firstly, the chosen model must operate in an unsupervised manner, a necessity due to the scarcity of labeled data in the maritime domain. Secondly, any trained model instance should provide a level of explainability, i.e., it must allow the user to understand and attribute the features that most significantly contribute to the identification of faults. Finally, the architecture must be as adaptable as possible: it should be domain-agnostic and capable of handling any type of multivariate time-series data without being specialized to only certain types or patterns of anomalies. Among the various candidates satisfying these conditions, we opted for an architecture that belongs to the family of MTAD-GAT models [50,51], which extract spatio-temporal information from multivariate time series by leveraging Graph Attention Networks (GATs) [52]. This choice is particularly justified in our context because shipboard machinery and their constituents can be represented as interacting sub-systems, and the relationships among these sub-systems are naturally modeled by graphs. Thus, we may flexibly define any system as a sub-graph of the overall machinery network. The architecture is depicted in Figure 5.

To train a model instance based on this architecture, a multivariate time series with *n* variables that has already been properly pre-processed is drawn from the Delta Lake’s silver layer. Before being fed to the model, it is split into potentially overlapping segments of equal length, *w*, as the proposed model operates in a sliding-window setting. Each of these n×w-dimensional segments corresponds to a single data point as far as the model is concerned. The end goal is to accurately predict whether the n×1-dimensional vector at the last timestamp of a window corresponds to a normal or fault state for the machine from which the multivariate time series was extracted. However, the DL model is not trained as a binary classifier.

Instead, a joint optimization strategy is followed, attempting to jointly minimize a reconstruction-based loss function and a forecasting-based loss function, $L_{r}$ and $L_{f}$, respectively. The idea behind this is to train the model to accurately predict upcoming measurements from the sensors installed on the machine while also capturing their data distribution. Then, newly arriving measurements can be evaluated on two axes: how close their values are to the trained model’s prediction and how likely it is that they have been generated by the distribution the model has learned. Based on the significance of the deviation for the reconstruction and forecasting process, a score is assigned to each timestamp. Finally, the state corresponding to each timestamp is identified as a fault or not, depending on whether the score is over or under a specified threshold, respectively.

At the start of the architecture lies a one-dimensional convolutional layer with unitary stride and dilation, whose purpose is the extraction of high-level information as a type of local feature engineering. This is mainly achieved due to the temporal invariance of one-dimensional convolutional layers, as they are able to detect features regardless of their shift in time, similar to how two-dimensional convolutional layers detect features in images. The size of the convolution kernel, denoted by κ, is one of the architecture’s hyperparameters. This layer’s output is $n\times \tilde{w}$-dimensional, where $\tilde{w}=w-\kappa$. Three copies of this output are made: the first copy is not further processed, while the other two pass through two parallel GATv2 layers, one spatial and one temporal. GATv2 layers are different from the original GAT layers introduced in [52] in that they are more expressive because their attention mechanism is not static but rather dynamic [53].

For the spatial layer, each variable of the time series is represented by a $\tilde{w}$-dimensional node in a graph, with the graph’s edges capturing the relationship between variables. This allows the GATv2 layer to detect multivariate correlations without any prior knowledge through the dynamic attention mechanism. Similarly, for the temporal GATv2 layer, each timestamp within a sliding window is represented by an *n*-dimensional node in a graph, with the graph’s edges now capturing temporal relationships. For both layers, the embedding dimension is equal to the input dimension, meaning that the total number of nodes, as well as their dimension, remains fixed. Therefore, the output of the spatial GATv2 layer is $n\times \tilde{w}$-dimensional, and the output of the temporal GATv2 layer is $\tilde{w}\times n$-dimensional. By transposing the latter and then concatenating both of them along with the unprocessed $n\times \tilde{w}$-dimensional copy of the convolutional layer’s output, a $3n\times \tilde{w}$-dimensional matrix is produced. This concludes the first half of the architecture, which essentially performs feature engineering. Each column of the final matrix corresponds to a 3n-dimensional feature vector at a given timestamp, which fuses the original input’s information with the spatio-temporal dynamic attention scores.

The second half of the architecture begins with a single LSTM layer with a hidden dimension *d*, which receives the feature matrix as its input. The purpose of this layer is to capture sequential patterns and long-term dependencies in the temporal dimension, a task at which recurrent neural networks and especially LSTMs excel. Using this layer’s output, the architecture then splits into two directions. The first corresponds to the reconstruction process, where the entire architecture so far is treated as an encoder, and a decoder is added at the end. This decoder consists of $N_{r}$ sequential LSTM layers with hidden dimension $d_{r}$ and a linear layer to map the LSTMs’ output to an n×1-dimensional vector. The second direction involves forecasting, where $N_{f}$ sequential LSTM layers with a hidden dimension $d_{f}$, followed by a linear layer, try to predict the *n* variables of the last timestamp of each window, without prior knowledge of them. To achieve this, each data point has to pass through the architecture twice, in parallel: once as it is for the reconstruction direction and once with the data corresponding to the last timestamp removed for the forecasting direction.

To train instances of this model, both $L_{r}$ and $L_{f}$ are calculated as the Root Mean Square Error (RMSE) between the model’s predictions and the actual measured values, although other loss functions have also been proposed for similar architectures [54]. This is a form of autoregression; therefore, the model is trained in an unsupervised manner, as required. The total loss function $L=L_{r}+L_{f}$ is minimized using Stochastic Gradient Descent (SGD), as implemented by the Adam optimizer. To avoid overfitting, dropout layers are inserted wherever applicable (i.e., between consecutive layers) with a probability of 20%. An additional regularization technique utilized is the introduction of a patience mechanism with limit *p*: the data points used for training are split into two sets, and only one of them is used to train the model by updating its parameters via backpropagation. The other set, usually called the validation set, is only utilized to evaluate the model’s performance during each training epoch. If the validation loss does not increase for *p* consecutive training epochs, then training is terminated and the model instance with the lowest achieved validation loss is saved.

With a model instance trained, a scoring function is necessary to assign scores to each timestamp’s values based on the ground truth and the model’s predictions. In addition, a mechanism to calculate a threshold is required. Suppose that the measured values at a given timestamp, *t*, are the elements of $u^{\left(t\right)}\in R^{n}$, and the corresponding predictions by the model are the elements of ${\hat{u}}_{r}^{\left(t\right)}\in R^{n}$ and ${\hat{u}}_{f}^{\left(t\right)}\in R^{n}$ through reconstruction and forecasting, respectively, then the label ${\hat{y}}^{\left(t\right)}$ for this timestamp is defined as

(1) $${\hat{y}}^{\left(t\right)}=\left\{\begin{array}{cc} 1, & \mathrm{if}\;S^{\left(t\right)}>\tau \\ 0, & \mathrm{otherwise} \end{array}\right.,$$

where $S^{\left(t\right)}$ is the score at timestamp *t* obtained from a scoring function $S\left(u^{\left(t\right)},{\hat{u}}_{r}^{\left(t\right)},{\hat{u}}_{f}^{\left(t\right)}\right)$, τ is the threshold, and a value of 0 or 1 corresponds to a normal or fault state, respectively.

As far as the scoring function is concerned, to ensure explainability for the final predictions, one may define a variable-wise score, $S_{i}$, as

(2) $$S_{i}=\omega_{i}\cdot \frac{\left|u_{i}-{\hat{u}}_{r,i}\right|+\gamma_{i}\left|u_{i}-{\hat{u}}_{f,i}\right|}{1+\gamma_{i}},$$

where $\omega_{i}$ is a weighting factor that controls the influence of the *i*-th variable on the overall score and $\gamma_{i}$ is a regularization parameter that modulates the impact of the forecasting error relative to the reconstruction error. If $\gamma_{i}>1$ or $\gamma_{i}<1$, the variable-wise score is influenced more by the forecasting error or by the reconstruction error, respectively. These parameters are useful in cases where domain expertise can be utilized to drive the fault detection process. For instance, if one knows beforehand that vibration measurements are more important than the environment’s temperature in the study of a motor, then the ω-value of the former can be set higher than the ω-value of the latter. Nevertheless, in cases where the data are scarce or such domain knowledge is not accessible, it is advised to start with $\omega_{i}=\gamma_{i}=1$ for all variables. Obviously, the overall score is obtained by summing over all variable-wise scores as

(3) $$S=\sum_{i=1}^{n}S_{i}=\sum_{i=1}^{n}\omega_{i}\cdot \frac{\left|u_{i}-{\hat{u}}_{r,i}\right|+\gamma_{i}\left|u_{i}-{\hat{u}}_{f,i}\right|}{1+\gamma_{i}}.$$

When it comes to the thresholding mechanism, ideally, thresholds would be derived utilizing domain knowledge. In its absence, the framework requires an automated method to derive thresholds that scales computationally well with the introduction of many machines, does not rely on labeled data, and is easy to re-execute whenever more training data become available. One such mechanism, proposed by [55], uses LSTMs for anomaly detection and is adopted in our framework with some modifications. Suppose there are *T* data points available for model training; their corresponding scores form the set $S=\left\{S^{\left(1\right)},\cdots ,S^{\left(T\right)}\right\}$. Depending on the scores’ values relative to the threshold, these scores can be split into two sets:

(4) $$\begin{array}{cc} S_{F} & =\left\{S\in S|S>\tau \right\},\\ S_{N} & =\left\{S\in S|S\leq \tau \right\}. \end{array}$$

By denoting the mean value and standard deviation of a finite set A by $\mu \left(A\right)$ and $\sigma \left(A\right)$, respectively, the threshold in this mechanism is given by

(5) τ=μ+zσ,

where z∈R is selected as a value for which the corresponding threshold maximizes

(6) $$\frac{1}{\left|S_{F}\right|}\cdot \left(2-\frac{\mu \left(S_{N}\right)}{\mu \left(S\right)}-\frac{\sigma \left(S_{N}\right)}{\sigma \left(S\right)}\right).$$

In other words, the threshold is chosen so that if all scores above it are removed from S, the greatest percent decrease in $\mu \left(S\right)$ and $\sigma \left(S\right)$ occurs, weighted by the inverse number of total faults to prevent classifying many timestamps in the training data as faults. It is noted that (6) is expected to be maximized by multiple values of *z*. Searching for one such value of *z* via brute force might be computationally expensive in real-world scenarios. Therefore, as a rule of thumb, it is suggested to explore values for *z* in the $\left(2,10\right]$ range, with a step of 0.5.

### 2.4. Evaluation and MLOps

Before a trained model instance can be deployed in production to perform inference on new incoming data using the scoring function of Equation (3) and a generated threshold, its performance must be properly evaluated, as long as labeled data are available for this purpose. Even though the F1-Score is the simplest metric for such evaluations, it treats each point of the time series independently, as if it were uncorrelated to the rest of the time series. Furthermore, faults often manifest not as isolated points but rather as consecutive sequences known as events. Point-wise metrics do not capture the continuity of anomalous events, thus potentially misrepresenting their severity and often leading to more pessimistic scores for the evaluated models [56].

To address these, a point-adjustment (PA) strategy, proposed by [57], has been adopted by numerous authors. The idea behind PA is simple: in real-world scenarios, if any point within an event is correctly classified, a human operator will be alerted and can thus examine the monitored device to identify the fault. Based on this, the model does not necessarily need to classify all points within an event as faults; if at least one point within the event is identified, then all points within the segment can be marked as correctly predicted before the calculation of the F1-Score. However, this metric, known as the PA F1-Score, has shortcomings of its own: it tends to overestimate model performance, and it has been demonstrated that a random classifier can outperform state-of-the-art architectures when evaluated using this metric [58].

In the presented framework, an alternative evaluation strategy is utilized that incorporates multiple metrics, as recommended in [59]. We propose using both the regular F1-Score and the PA F1-Score for evaluation, which should ideally converge to similar values. Additionally, a third F1-Score termed the brute-force (BF) F1-Score, is calculated using a threshold obtained by a brute-force search across all possible threshold values to maximize the F1-Score. The argument in favor of this is that the previously mentioned scores evaluate not only the DL architecture but also the thresholding method given by Equation (5) [60]. The BF F1-Score evaluates the ability of the model itself to properly score the timestamps in a way that can successfully separate faults from normal values. Besides these metrics, the delay in detecting each event is also considered, with penalties applied for any events that remain completely undetected. Finally, training and inference times are reported, as the capability to retrain models within a limited timeframe and to process incoming measurements promptly is vital for industrial applications.

**Figure 6:**
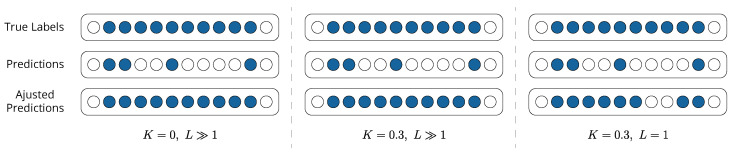
Depiction of how the proposed PA%K-L protocol can recreate the original point-adjustment strategy (**left**) and the PA%K protocol (**middle**) while also allowing for control of the number of adjusted points (**right**).

Specifically regarding the PA F1-Score, a stricter version called PA%K-L is proposed, inspired by the PA%K protocol [58]. Under the PA%K protocol, point adjustment is applied to all *N* points of an event only if the model correctly identifies more than K×N of them as faults, where 0≤K≤1. In our protocol, we introduce an additional parameter, *L*, which controls the number of points that undergo adjustment within an event. In particular, *L* represents the number of nearest-neighbor points around each correctly identified fault that are adjusted. The example shown in Figure 6 demonstrates how this protocol works compared to PA and PA%K. In all three cases, the event consists of 10 timestamps, and the model manages to predict only 4 of them. The original PA is obtained by setting K=0 and L≫1, in which case the entire event is considered correctly predicted by the model. For values of K≥0.4, the PA%K protocol dictates that the event is undetected, and therefore no point adjustment is performed. However, for K=0.3 and L≫1, the PA%K protocol coincides with regular PA. Nonetheless, when setting L=1, only one point around each correctly identified fault is adjusted, meaning that the adjusted predictions lead to an F1-Score that is higher than the non-adjusted one but lower than its PA version.

By this point, the specifics of model training and evaluation have been thoroughly discussed. However, it is important that in a real-world application, they adhere to standard MLOps practices. Model instances must be registered and tracked through all stages of their lifecycle. During the training and hyperparameter tuning phases, models should remain in a “development” stage where they can be iteratively adjusted and optimized. Once training is complete, models transition to a “staging” stage for evaluation and testing. If they meet predefined performance criteria with respect to the aforementioned evaluation metrics, they then advance to the “production” stage, where they are deployed to perform inference on new incoming data. Orchestrating this process in a scalable and automated manner is crucial, especially when monitoring numerous machines.

The open-source platform that is selected to orchestrate the DL workflows in our framework is MLflow [61,62], developed and managed by Databricks. During model training, an MLflow experiment is defined to track all relevant metrics and artifacts using MLflow’s tracking server. Specifically, the total training time, along with the loss function’s value at the end of each epoch, is logged. Additionally, all model hyperparameters are archived as JSON files for reproducibility, and the calculated threshold is stored for use during evaluation and inference. Finally, the trained model instance is saved in MLflow’s model registry, along with all the details of the environment in which it was developed and trained. Once hyperparameter tuning is complete, i.e., after training and comparing the loss function’s values for different model instances, one or more of them transition to the next stage.

For model evaluation, a different MLflow experiment is initiated, where all the discussed evaluation metrics are logged, including the average time taken to perform scoring on a single timestamp’s measurements. If, after evaluation, a model instance surpasses the performance of the currently deployed production model in terms of the evaluation metrics, it is then promoted to production, while the previous instance is archived. Naturally, being able to compare model instances and determine which is superior is not straightforward when multiple metrics are involved. Hence, the following composite metric is proposed:

(7) $$\begin{array}{cc} F=\frac{1}{\lambda_{1}+\lambda_{2}+\lambda_{4}}\cdot \max(0,\; & \lambda_{1}F_{1}+\lambda_{2}F_{1}^{*}-\lambda_{3}\left|F_{1}-F_{1}^{*}\right|\\ \; & +\frac{\lambda_{4}}{N_{E}}\sum_{i=1}^{N_{E}}\frac{1}{1+\theta_{i}d_{i}}-\lambda_{5}\frac{N_{\mathrm{und}}}{N_{E}}), \end{array}$$

where $F_{1}$ is the model’s regular F1-Score, $F_{1}^{*}$ is the corresponding PA F1-Score obtained using the PA%K-L protocol, $N_{E}$ is the number of events in the time series, $N_{\mathrm{und}}$ is the number of undetected events, and $d_{i}$ is the model’s delay in identifying the *i*-th event. Additionally, $\lambda_{1},\cdots ,\lambda_{5}\geq 0$ are tunable hyperparameters that regulate the contribution of each term to the overall score, and $\theta_{i}\geq 0$ are event-dependent factors that quantify the importance of the *i*-th event’s identification delay. It should be noted that the delay can be defined in terms of either actual timestamps or sliding windows, depending on the problem at hand. The definition of what constitutes an event is also problem-dependent.

The first two terms of F define its linear dependence on the two versions of the F1-Score, with the $\lambda_{1}$ and $\lambda_{2}$ coefficients regulating each term’s contribution. To place more focus on the regular F1-Score than the PA one, it suffices to choose suitable values for the coefficients, satisfying $\lambda_{2}<\lambda_{1}$. However, in addition to this, it may also make sense to penalize significant discrepancies between the two. This is where the third term comes in, with $\lambda_{3}$ quantifying the impact of these discrepancies on the overall score. The fourth term is related to the model’s delays in identifying events. Significant delays are equivalent to large values of $d_{i}$ and therefore lead to minor contributions to the composite metric, despite the successful identification of the corresponding events. To make the impact of the delays event-dependent, the $\theta_{i}$ factors are introduced. Thus, an event with higher significance than others (e.g., an event that is known to lead to the complete breakdown of a machine) can have a higher θ-value assigned to it. Of course, such assignments require explicit domain knowledge; therefore, it makes sense (at least initially) to set $\theta_{i}=\theta ,\forall i\in {\left\{i\right\}}_{i=1}^{N_{E}}$ in order to avoid biases that do not arise from deep insights into the system’s physics. Finally, the fifth term is introduced to explicitly penalize the unsuccessful identification of an event. Even though the fourth term’s contribution would be zero in these cases (since d→∞), it is sensible to impose a penalty by subtracting from the overall score, instead of simply “not adding” anything to it.

In the best-case scenario, the model manages to classify all points of the time series correctly, in which case $F=F^{*}=1$, $N_{\mathrm{und}}=0$, and $d_{i}=0,\forall i\in {\left\{i\right\}}_{i=1}^{N_{E}}$, resulting in F=1. In the worst-case scenario, where the model fails to identify any of the time series’ anomalies, $F=F^{*}=0$, $N_{\mathrm{und}}=N_{E}$ and $d_{i}\to \infty ,\forall i\in {\left\{i\right\}}_{i=1}^{N_{E}}$ hold, leading to F=0 due to the max function that ensures no negative scores arise. Closing the discussion on the evaluation of model instances as part of their lifecycle, it is important to stress that, apart from the composite metric of Equation (7), an additional requirement for a model instance to transition to production is to achieve a BF F1-Score that is higher than a pre-determined threshold, which is dataset-dependent.

Ultimately, the model instances that have advanced to the production stage are deployed for inference on new data. Since these new data are stored persistently in the lakehouse, with the process already described, they are available for labeling by a domain expert (e.g., an engineer on the vessel). In this way, normal segments can be used for future retraining of the model instances, and new evaluation datasets can be constructed, containing a mixture of normal data and faults. The development–staging–production cycle can then begin anew, with existing model instances being refined to achieve better performance or new model instances replacing them altogether.

## 3. Experiments

With all aspects of the framework thoroughly discussed, this section demonstrates its practical application through three different case studies. One of the challenges in the field of fault detection is the scarcity of publicly available datasets, as many studies rely on proprietary data, limiting the reproducibility of their experiments. Moreover, some of the datasets frequently used for benchmarking fault detection models have been criticized [63] for issues such as trivial faults or incorrect labels. While there are no guarantees that they are universally ideal for benchmarking, the open-source datasets selected for the following case studies are believed to effectively demonstrate the capabilities and adaptability of the proposed framework in a transparent and reproducible manner.

To simulate a realistic scenario as accurately as possible, where (at least in the beginning) no prior domain expertise is available, we set $\omega_{i}=\gamma_{i}=1,\;\forall i\in {\left\{i\right\}}_{i=1}^{n}$ for the variable-wise score in Equation (2). For the same reason, we choose $\theta_{i}=1,\;\forall i\in {\left\{i\right\}}_{i=1}^{N_{E}}$ for the composite metric in Equation (7). Additionally, $F_{1}^{*}$ is calculated by selecting K=0.8 and L=2 for the PA%K-L protocol so that only minor point adjustments are performed. This implies that $F_{1}\approx F_{1}^{*}$ is expected. Therefore, we set $\lambda_{3}=0.2$ to penalize possible discrepancies. For the remaining λ hyperparameters, we set $\lambda_{1}=0.5$ and $\lambda_{2}=0.35$ to place more weight on the regular F1-Score, and $\lambda_{4}=0.15$, $\lambda_{5}=0.1$ for the delay penalties.

### 3.1. Showcasing Performance and Explainability: EFDC Dataset

The first case study corresponds to what is known as the Electrical Fault Detection and Classification (EFDC) dataset [64], which contains a single multivariate time series of synthetic data. In [65], it is referenced as the Transmission Line Faults dataset and is given a grading of II among datasets curated for the purposes of PdM in the energy sector. Out of its six variables, three correspond to currents and are denoted by $I_{a}$, $I_{b}$, and $I_{c}$, and three correspond to voltages and are denoted by $V_{a}$, $V_{b}$, and $V_{c}$. The dataset contains a total of 12,000 timestamps, 5496 of which correspond to faults, split across five distinct events. Although electrical measurements are generally utilized in performing condition monitoring of machinery, with novel techniques being introduced in the modern literature [66], the main reason for choosing this “easy” dataset as our first case study is to demonstrate the framework’s performance, explainability, and evaluation aspects.

To this end, the first 400 timestamps were reserved for model training. To simulate the process performed after ingestion in the lakehouse, a min-max scaler was trained on the training subset of the data and then applied to it. Subsequently, the data were organized in windows of size w=100 with unitary stride and fed to the architecture for training. All model instances were trained by reserving 10% of the training subset for validation and using a patience threshold p=10. After training some instances and comparing their validation losses, the model instance saved for evaluation had the following hyperparameter values: κ=7, d=150, $N_{r}=N_{f}=2$, $d_{r}=350$, and $d_{f}=150$. The training time was approximately 0.1 s per training epoch. All benchmarks mentioned for training and inference times were acquired using a single RTX 4070 GPU (Nvidia, SCL, CA, USA).

Following the calculation of a threshold using the framework’s automatic thresholding mechanism, the remaining 11,600 samples were transformed using the trained min-max scaler and fed into the trained model instance for evaluation. The average inference time per timestamp, including scoring and predicting faults based on the threshold, was 0.1 milliseconds. The F1-Score achieved was 97.68% without PA and 98.01% with PA, and all five events were successfully detected with no delays. Plugging these values into Equation (7) resulted in F=98.07%. At first glance, it might seem counterintuitive that the resulting F-score was higher than both the F1-Score and the PA F1-Score; however, one must keep in mind that the composite score also considers the delay for the identification of each event, which is rewarded when small or zero. For example, if one of the five events had a delay of 1 timestamp, the corresponding result would be F=96.58%, i.e., smaller than both F1-Scores.

**Figure 7:**
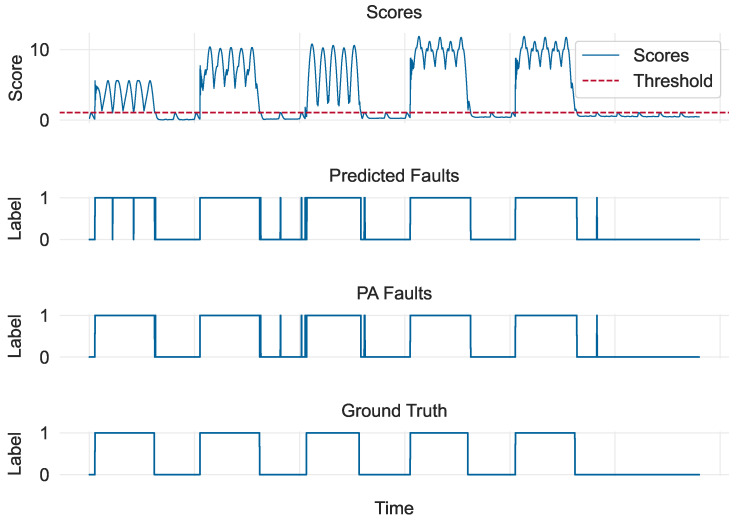
Graphs depicting the model’s scores for the evaluation data points (**top**), the corresponding predicted anomalies with and without PA (**middle**), and the ground truth based on the dataset’s labels (**bottom**).

The overall scores for the evaluation subset obtained using Equation (3) are depicted in the first graph of Figure 7. The second and third graphs show the corresponding predicted anomalies without and with PA, respectively. The ground truth is depicted in the last graph. It is evident that the model performed remarkably well; however, this was mainly because the faults in the dataset were relatively easy to identify, which is why the scores corresponding to faults are an order of magnitude higher than those corresponding to normal data. Interestingly, the effect of performing PA can be seen by focusing on the first event in the middle graphs: even though the model failed to identify some timestamps as faults, the PA%K-L protocol performed adjustments to the *L* nearest neighbors of each correctly identified fault because the *K*-criterion was met, thus making the unidentified faults of the first event disappear in the PA version.

**Figure 8:**
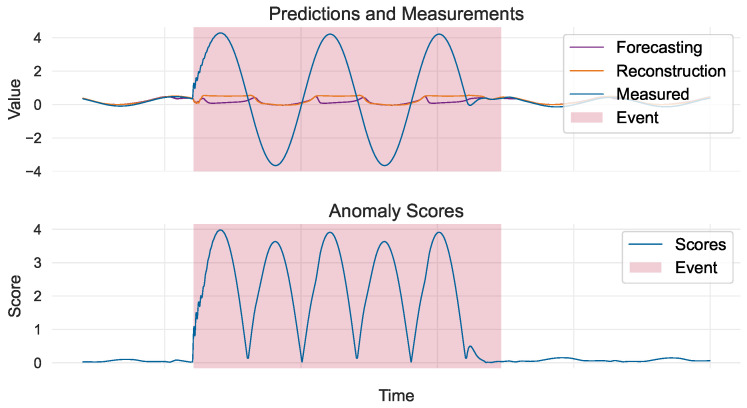
Example of reconstruction and forecasting of the $I_{c}$ variable before, during, and after the fifth event, compared to the actual measured values.

Apart from discussing the model’s overall performance and how evaluation works, it is also worth showcasing its explainability by investigating the contribution of each dataset’s variable to a timestamp’s overall score. In the example shown in Figure 8, we demonstrate how the $I_{c}$ variable contributed to the detection of the fifth event. Before and after the event, the measured values were almost identical to those predicted by the model via reconstruction and forecasting. However, for the duration of the event, the model struggled to correctly forecast or reconstruct the measured values—as expected, since they correspond to faults. This produced large variable-wise scores, which contributed to the overall score and led to the detection of the event.

**Figure 9:**
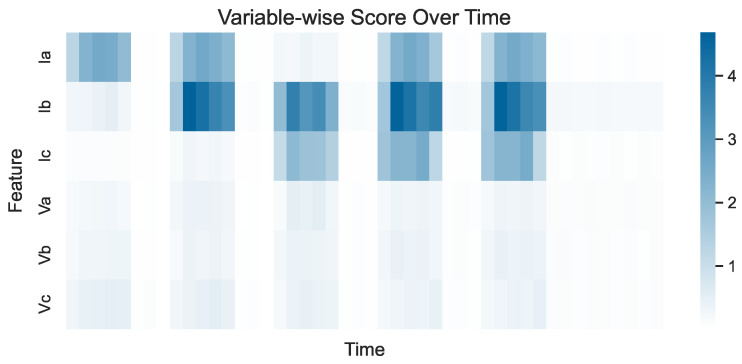
Aggregated variable-wise scores for all features across all timestamps, with bins of width Δt=250.

Nevertheless, plots like the one shown in Figure 8 do not enable a comparison of the variable-wise scores across different variables. To achieve this, one may construct heatmaps of variables versus time (measured in timestamps), where the color intensity corresponds to the variable-wise score, as shown in Figure 9. Note that the scores have been aggregated through time in bins of width Δt=250 (measured in timestamps) to make the plot more easily readable. Inspecting the heatmap in Figure 9 can lead to several conclusions. Firstly, the five events of the dataset can be discerned as five vertical strips over the time axis. Additionally, the variables that drive the detection of events are easily recognizable. For instance, $I_{b}$ and $I_{c}$ are mainly responsible for the detection of the third event, while all current variables appear to be responsible for the detection of the fourth and fifth events. Lastly, it can be seen that the overall contribution of the voltage variables appears to be small compared to the contribution of the current variables.

**Figure 10:**
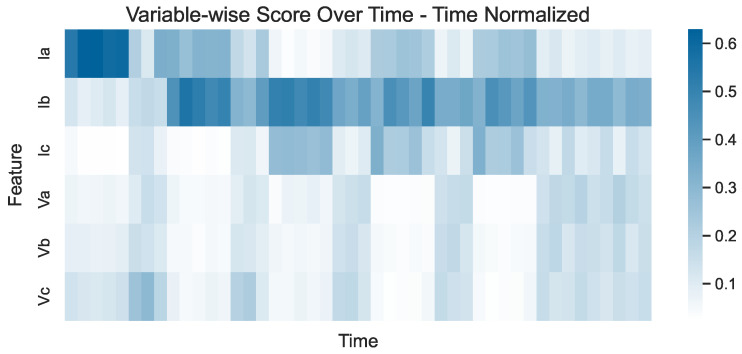
Aggregated variable-wise scores for all features across all timestamps, with bins of width Δt=250; time-normalized.

To further illustrate the last two observations, the same heatmap can be constructed, this time depicting time-normalized variable-wise scores. In the time-normalized plot shown in Figure 10, the variable-wise scores are adjusted so that their sum at each timestamp equals 1. This diminishes the emphasis on when faults occur and instead highlights which features are comparatively more influential at any given timestamp. For instance, in the plot in Figure 10, it can be seen that the main variable driving the detection of the first event is without a doubt $I_{a}$, even though its absolute variable-wise score does not appear to be very high in the plot in Figure 9 (especially when compared to the variable-wise scores obtained for other variables during other events). Additionally, the voltages are clearly not influential for detecting the events, especially the fourth and fifth events, where their contribution is practically zero compared to the currents.

**Figure 11:**
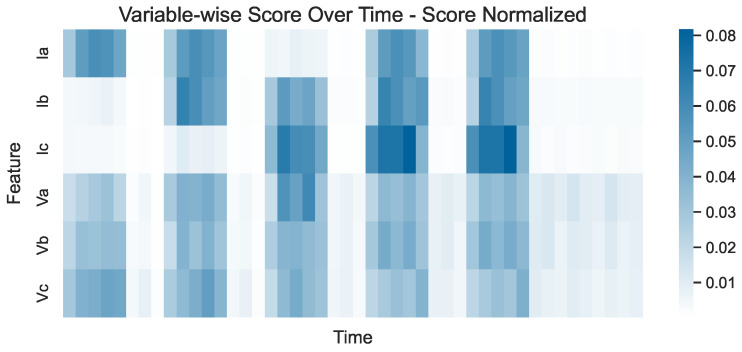
Aggregated variable-wise scores for all features across all timestamps, with bins of width Δt=250; score-normalized.

Of course, this does not imply that the voltages are not affected by the events. If the scores are normalized so that the sum for each variable is equal to 1 across all timestamps, the score-normalized plot shown in Figure 11 is constructed. There, it can be clearly observed that all voltage variables are affected by the faults since their corresponding scores are higher during the events and lower for normal values. On the other hand, $I_{c}$ is clearly unaffected by the first two events, while $I_{a}$ and $I_{b}$ are unaffected by the third and first events, respectively—an observation that cannot be made when focusing on the former two plots. This suggests that each of these plots highlights different aspects of the model’s performance and all three should be used in conjunction during evaluation to avoid misinterpretations or missing valuable insights.

### 3.2. Benchmarks on Real Machinery Data: CWRU Dataset

In the second case study, the chosen dataset is related to motor bearings, which are often monitored on vessels using traditional methods, and comes from the Bearing Data Center at the Case Western Reserve University (CWRU) [67]. The data correspond to acceleration measurements gathered from experimental tests on a 2 HP electric motor. The dataset contains three variables: DE (Drive End), FE (Fan End), and BA (Base), each of which indicates the position of the corresponding accelerometer in relation to the motor bearings. However, not all data files include these three variables; most contain both DE- and FE-type time series, only a few include all three, and eight data files contain only DE-type time series. To create a uniform dataset that contains as many data files as possible, the latter eight data files are discarded, and the BA variable is disregarded, resulting in a dataset comprising 153 data files with two variables each.

The data files correspond to four distinct classes—1730, 1750, 1772, and 1797—based on the speed of the motor (in RPM) during the data collection process. This implies that four distinct model instances are required for evaluation. For each class, there are four categories of files: 1 data file containing time series for normal bearings measured at 12 kHz, 26 data files containing time series for bearings with single-point DE defects measured at 12 kHz (13 files) and 48 kHz (13 files), and 11 data files (12 for the class of 1797 RPM) containing time series for bearings with FE defects measured at 12 kHz.

With the exception of the normal data, each file corresponds to a unique fault identified by its diameter, which can be $0.007^{\prime \prime }$, $0.014^{\prime \prime }$, or $0.021^{\prime \prime }$, and type, depending on whether it was introduced in the inner raceway (IR), the rolling element (i.e., ball (B)), or the outer raceway (OR). The original CWRU dataset also includes $0.028^{\prime \prime }$ faults; however, these are present in the eight files that were discarded for this study because they contain only a single time series. Regarding OR-type faults, further differentiation is made with respect to the position relative to the load zone, which is centered at 6:00. Based on this, a file may correspond to an OR centered fault (at 6:00-OR@6), an OR orthogonal fault (at 3:00-OR@3), or an OR opposite fault (at 12:00-OR@12).

The data files are provided in .mat format. Motivated by the fact that the original files contain some inconsistencies in their contents and redundancies in their metadata, we converted them to .npz format (which is more common in DL tasks due to the widespread use of Python) and fixed the inconsistencies. This “corrected” dataset can be found in a public GitHub repository (https://github.com/srigas/CWRU_Bearing_NumPy, accessed on 10 August 2024), along with a detailed changelog and information about the file naming conventions so that it can be readily utilized in future works similar to the one presented here.

Apart from these corrections, additional wrangling was necessary before applying our framework. In the past, the dataset was mainly used for classification or fault diagnosis tasks because each data file comprises an entire event. To utilize the dataset for fault detection, we split each of the normal-data time series into two segments: the first segment was used for training model instances, while the second segment was appended at the beginning of all fault data files of the corresponding RPM class. Table 1 shows the number of timestamps available for the training and evaluation of the model instance for each class.

Once this initial pre-processing was performed—a task that can be thought of as simulating the edge pre-processing steps discussed in the previous section—the data for each of the four normal data files were min-max scaled and organized in windows of size w=100 with a stride of 50 timestamps. As in the case of the EFDC dataset, 10% of the training data were reserved for validation, and the patience threshold was set to p=10. More than 20 instances were trained for each RPM class, with an average time of 1.9 s per training epoch. This is an order of magnitude higher than the EFDC dataset, which contained more variables due to the significantly higher number of data points available in the CWRU dataset. The hyperparameters for the four trained instances with the lowest validation losses can be seen in Table 2.

**Figure 12:**
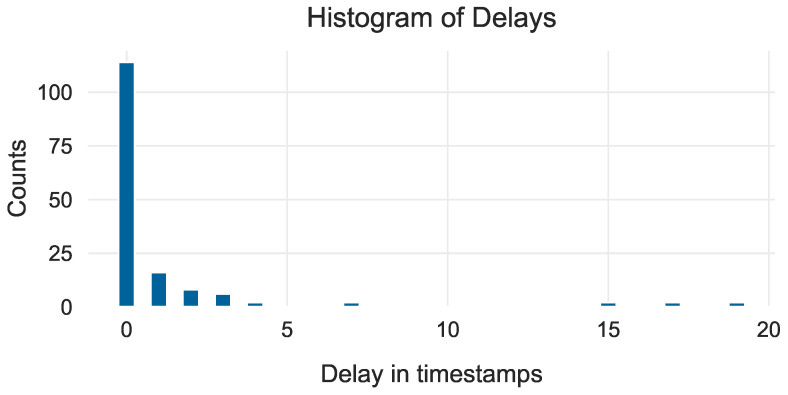
Histogram depicting the delay of event identification (measured in timestamps) for all data files in the CWRU dataset.

Once a threshold was calculated for each RPM class, the evaluation data were scaled and passed to the corresponding model instance. During the evaluation, the average inference time per timestamp, including scoring and predicting faults based on the threshold, was 0.09 milliseconds. Interestingly, despite using only a portion of the normal data for training and reserving the rest for evaluation, almost all faults were identified with practically no delay, as highlighted in the histogram in Figure 12. This is a noteworthy feat because in this work, the delays are measured in terms of timestamps instead of time windows (in which case all events would have a delay of 0 since w=100). Nonetheless, there are cases where the delay is higher than 0, sometimes even higher than 10 timestamps.

**Figure 13:**
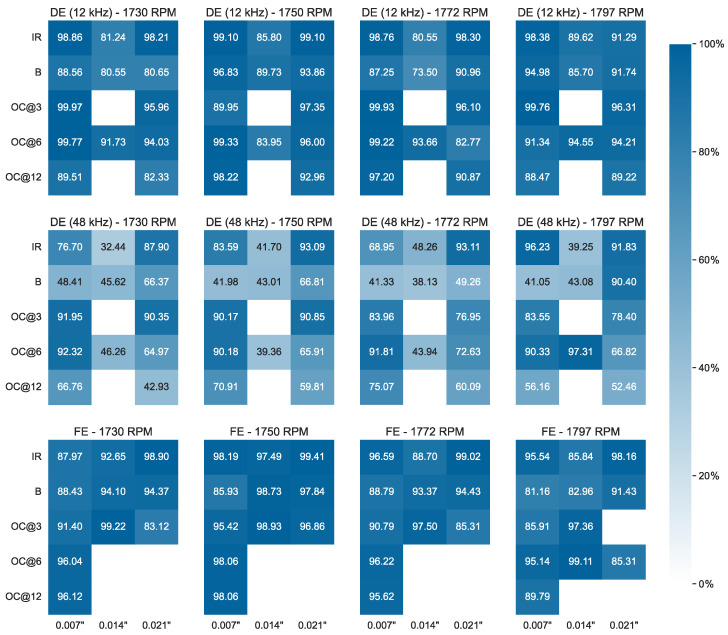
Evaluation results in terms of the F metric for the four model instances trained on the CWRU dataset’s baseline data. Each row corresponds to a different category, while each column corresponds to an RPM class, i.e., a unique trained model instance.

To gain a better understanding of this, one may turn their attention to the detailed results per data file in terms of the F metric, as depicted in Figure 13. Note that not all combinations of fault diameters and fault types are present in the CWRU dataset, which is why several portions of the heatmaps in Figure 13 are blank. In general, no significant differences can be seen between the four trained model instances, with their overall performance being more than satisfactory: for 119 out of 149 files, the final score was over 75%. The best results can be seen in the FE category of faults, while in the DE (12 kHz) category, it is evident that the models were generally less effective at identifying faults of diameter $0.014^{\prime \prime }$ compared to those with diameters of $0.007^{\prime \prime }$ or $0.021^{\prime \prime }$. However, among the 30 data files for which F was lower than 75%, it is no coincidence that all of them belong to the DE (48 kHz) category. Notably, these are also the cases where the aforementioned high delays were recorded, with a maximum delay of 19 timestamps corresponding to the IR-$0.014^{\prime \prime }$ fault of the 1730 RPM class. This data file represents the case with the minimum F-score of 32.44%.

The reason the performance on these files was relatively low for all model instances is related to the frequency at which the measurements for these data were collected. In particular, these data files were obtained from 48 kHz measurements, while the data for all other files—including those containing the normal data used for training—were collected at 12 kHz. If the data were transformed into frequency spectra using a Fourier transformation, the features extracted from the 48 kHz data would probably not differ significantly from those extracted from the 12 kHz data (perhaps with the exception of the appearance of some additional harmonics), and therefore the performance of a different model would not be significantly affected. In this case, however, we opted to work with an architecture that generally processes raw time series, which undergo only minor pre-processing via min-max scaling, in order to demonstrate not only the generalizability but also the limitations of the framework when applied with no prior domain expertise.

**Figure 14:**
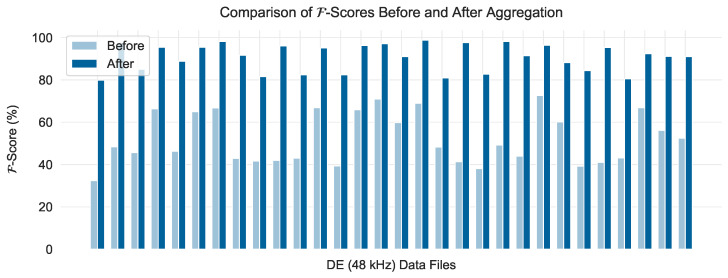
Paired bar chart of F for DE (48 kHz) data before (light blue) and after (dark blue) aggregation to reduce the effective sampling frequency.

Nonetheless, these results do not indicate that a separate model instance would have to be trained in order to achieve more satisfactory results for the DE (48 kHz) data files. Instead, one may simply perform a median aggregation with non-overlapping windows of four timestamps to reduce the effective sampling frequency from 48 kHz to 12 kHz. By performing this simple pre-processing technique before scaling the data and splitting them into sliding windows, the resulting F-scores obtained by the same model instances become comparable to those achieved for the DE (12 kHz) or FE categories. This is demonstrated in Figure 14, where the evaluation results for the 30 data files that originally scored under 75% are depicted before (light blue) and after (dark blue) the aggregation technique. Note that the data files depicted are grouped by their RPM class and ordered in the same way as in Figure 13. After the aggregation, the lowest increase in the F-score for these files is +23.77%, while the highest increase is +56.32%, achieved for the B-$0.007^{\prime \prime }$ fault of the 1772 RPM class.

An important remark should be made before closing the discussion on this case study. Naturally, it is not sensible to simply dismiss three-fourths of the available data, as was done when performing the aggregation technique to effectively reduce the frequency of the original data. This is only a workaround to demonstrate how easily the framework can adapt to address such problems, which is also the reason we chose to present the “bad” results in the first place. One such problem could occur, for instance, in a scenario where new sensors are installed on a machine, with a frequency higher than that of their predecessors. In this case, this workaround could be a transitional solution until enough data are collected to start training new, more efficient model instances.

### 3.3. Inter-Domain Generalizability: SKAB Dataset

The final case study utilizes the Skoltech Anomaly Benchmark (SKAB) dataset [68], which contains real measured data obtained from a testbed designed for water circulation experiments. The dataset contains eight variables per available timestamp: two RMS values derived from accelerometers, the current and voltage of the electric motor connected to the testbed, the temperature of the engine body as well as the temperature of the fluid in the circulation loop, the pressure in the loop after the water pump, and the circulation flow rate of the fluid.

While not as commonly studied as motors or electrical machines, problems related to water circulation also occur in vessels, such as in HVAC or ballast water treatment systems. The previous datasets predominantly focused on specific types of machinery issues, such as bearing or electrical problems. In contrast, the SKAB dataset offers an opportunity to test the framework’s generalizability across a broader spectrum of faults, including those not directly relevant to marine applications. For this study, we specifically selected 14 data files from the category labeled as other within the SKAB dataset. This category comprises experiments that simulate a range of faults, from rotor imbalances that resemble Dirac delta functions to fluid leaks and additions, which are not necessarily typical for any single type of marine machinery or system.

Similar to the CWRU dataset, each file in the SKAB dataset documents a single event; however, in this case, measurements from the normal state are also included. A subset of these normal readings was set aside to train a separate model instance for each file. The provided time series originally designated for training was not utilized because its measurements appear to significantly differ from those labeled as normal in the fault-containing files. For example, the RMS value for one of the accelerometers in the fault-free time series is an order of magnitude higher than its value in the normal data for some of the files that include faults. This is an issue known to the creators of the SKAB dataset, and their benchmarks for fault detection were obtained using the same approach [68].

**Figure 15:**
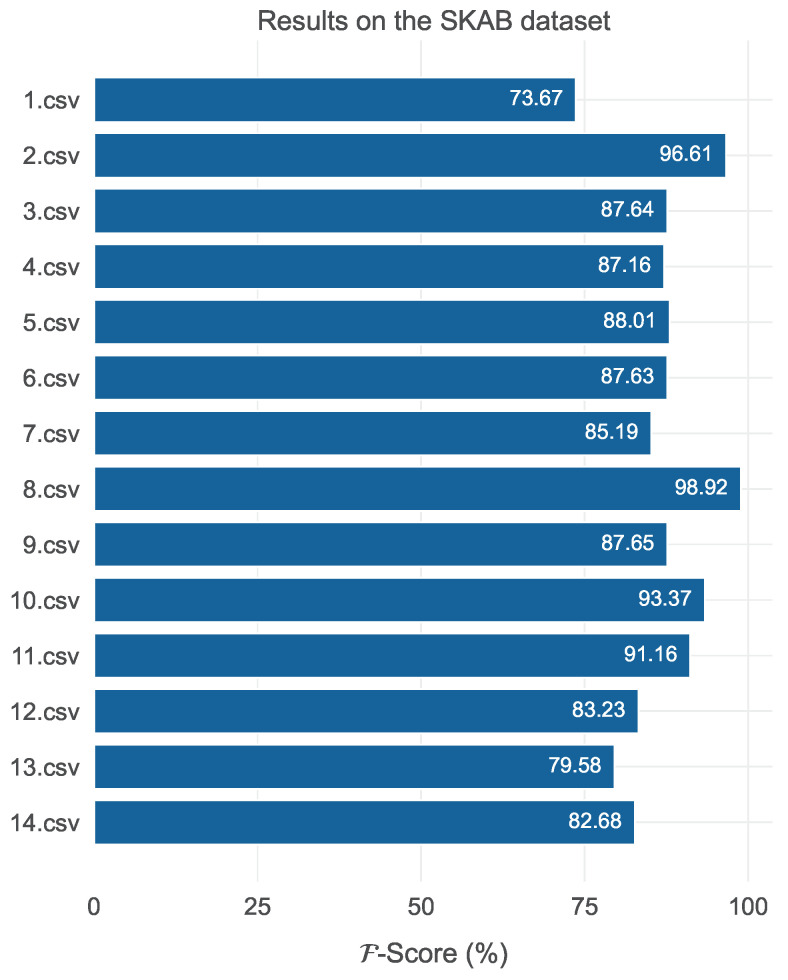
Horizontal bar plot showing the F-scores for each data file in the SKAB dataset.

The portion of data used for the training of a model instance per data file was approximately 25% of the file’s data. As in the previous cases, the training data were used to train a min-max scaler, which was later utilized to transform the evaluation data. Additionally, the data were arranged in windows of size w=30 with unitary stride, and 10% of the training data were reserved for validation. The patience threshold was again set to p=10, while all trained model instances shared the same hyperparameters: κ=7, d=200, $N_{r}=2$, $N_{f}=3$, $d_{r}=250$, and $d_{f}=250$. With an average training epoch of 0.1 s and an average inference time per timestamp equal to 0.08 milliseconds, the evaluation of the models on the 14 data files in terms of the F metric can be seen in Figure 15.

With the exception of the 1.csv file, all F-scores were higher than 79%. While these results are more than satisfactory, especially when compared to the current benchmarks on the SKAB dataset, there is a chance that they are not indicative of the model’s true performance and that they represent underestimations. The reason for this is related to the labeling process. According to the current documentation for the SKAB dataset, only the actual fault state is labeled as such, while the transition from the normal to the fault state is not labeled as a fault. For this reason, several model predictions evaluated as false alarms led to an overall decrease in the F1-Score (and by extension, the F-score). In a real-world scenario, these false alarms would be extremely valuable, as they would correspond to timely alerts of an upcoming fault.

## 4. Conclusions

In this study, we presented a novel end-to-end solution for performing fault detection in multivariate time series extracted from monitored shipboard machinery. At the edge, the framework collects data from DAQs communicating via MQTT, processes them through Apache Kafka, and performs basic pre-processing before uploading them to a cloud storage service. Although the data are collected as streams, their upload to the cloud is orchestrated as a batch process to prevent overloading the vessel’s network. Once in the cloud, the data undergo a post-upload feature extraction process, which occurs in multiple stages within a Delta Lake architecture, orchestrated by Databricks and other cloud services. The extracted features are used to initially train and subsequently feed into an MTAD-GAT-type neural network for inference. Importantly, the inference results are not delivered as a “black-box” output; the attention mechanism inherent in GATs allows us to quantify the importance of each feature and its contribution to the model’s final output. Furthermore, we developed a composite metric to evaluate all DL model instances, mainly focusing on the F1-Score, the PA F1-Score as defined by the PA%K-L protocol, and the delays in identifying events within the time series. The lifecycle of each model instance is managed by MLflow, which is fully integrated into the Databricks ecosystem.

Using the DL architecture and the evaluation metric of the proposed framework, experiments were performed on three datasets related to PdM, one of which was modified to correspond to a fault detection task. To simulate the cloud pre-processing step, basic transformations were applied to the time series; however, they were not dataset-specific but rather universal for all three datasets. In this way, the adaptability of the framework without requiring explicit domain knowledge was demonstrated, as the trained model instances achieved satisfactory results in all cases. Notably, the worst performance of the model instances was observed for the SKAB data files, although the lowest score obtained for one of its files was 73.67%. Moreover, the explainability of the model via its scoring mechanism was showcased through the variable-wise scores available at each predicted timestamp, either raw, score-normalized, or time-normalized. Finally, as far as cost-efficiency is concerned, it is worth noting that in the presented experiments, we performed inference as part of the model instances’ evaluation for 11,600 timestamps in the EFDC dataset (six variables), 33,825,995 timestamps in the CWRU dataset (two variables), and 11,582 timestamps in the SKAB dataset (eight variables). If, instead of a custom-deployed model, an anomaly detection model was used as a service from a major cloud provider, the corresponding inference cost would be USD 67,814.25 [69].

If this framework were widely adopted within the marine industry as part of its ongoing digitalization efforts, the benefits would extend beyond increased safety for the crew and cost savings for stakeholders through PdM. Such adoption would also significantly impact the scientific community. On one hand, widespread adoption could motivate further research in the field, potentially leading to the development of novel DL architectures or alternative data collection and processing procedures. On the other hand, it could promote the curation and sharing of more open datasets, which are currently scarce. A practical approach to achieving this would be by utilizing the dashboard application used by the crew and stakeholders to consume the results of the DL models: after an engineer on board performs an inspection of the machinery’s status, they could annotate time-series segments immediately after model inference. This process would generate numerous labeled datasets relevant to fault detection tasks with high-frequency data, which could serve as valuable benchmarks across the entire domain. Such a resource would not only enhance the accuracy and robustness of future models but also establish a standard for evaluating fault detection systems within the industry.

Moving forward, as far as future research related to this topic is concerned, we believe that the framework could greatly benefit from current progress in large language models (LLMs) with retrieval-augmented generation (RAG). For instance, when new faults are detected, the LLM can automatically retrieve relevant information from past incident logs, maintenance records, or similar fault instances. It could then generate explanations or hypotheses about the likely causes of the fault, based on patterns or similarities found in the retrieved data. This process can help in quickly identifying potential recurring issues. Additionally, another direction for research could be the integration of domain expertise into the fault prediction process. In the presented framework, this is enabled through the introduction of adjustable hyperparameters in the variable-wise score and the composite evaluation metric. However, enriching the DL architecture itself with system-related information—such as incorporating driving equations in the loss function, similar to how physics-informed neural networks use differential equations—could significantly enhance its performance. Moreover, model-based approaches tend to perform well for systems with well-understood mechanics; therefore, combining AI techniques with first-principle-based models could offer the most robust solution, leveraging the strengths of both approaches [70].

## Figures and Tables

**Table 1:** Timestamps in CWRU for training and evaluation.

RPM	Training	Evaluation
1730	242,821	9,493,230
1750	242,531	9,495,364
1772	241,951	9,378,347
1797	121,969	5,459,054

**Table 2:** Architecture hyperparameters for CWRU.

RPM	κ	d	$N_{r}$	$N_{f}$	$d_{r}$	$d_{f}$
1730	9	200	3	3	250	250
1750	7	250	3	4	350	200
1772	9	200	3	4	250	250
1797	7	200	3	3	300	250

## Data Availability

All experiments conducted in this study utilized publicly available open datasets. All data sources are acknowledged within the text and referenced in the bibliography. As no new data were generated, additional access requests are not applicable.
